# Association between ideal cardiovascular health and bowel conditions among US adults

**DOI:** 10.3389/fnut.2024.1473531

**Published:** 2024-11-07

**Authors:** Yiwen Wang, Zhigang Wang

**Affiliations:** Xi’an International Medical Center Hospital Affiliated to Northwest University, Xi’an, China

**Keywords:** chronic diarrhea, chronic constipation, fecal incontinence, Life’s Essential 8, ideal cardiovascular health, NHANES

## Abstract

**Objective:**

The aim of this study is to explore the relationship between ideal cardiovascular health (CVH), as assessed using the Life’s Essential 8 (LE8), and bowel conditions.

**Methods:**

This cross-sectional study selected 11,108 participants aged ≥20 years from 2005 to 2010 National Health and Nutrition Examination Survey. LE8 scores (range 0–100) were measured according to American Heart Association definitions and were divided into health behavior and health factor scores. Bowel conditions including chronic diarrhea, constipation, and fecal incontinence were diagnosed by the Bowel Health Questionnaire. Weighted logistic regression and restricted cubic spline models were used for correlation analysis.

**Results:**

Logistic regression results showed that LE8 scores were negatively associated with chronic diarrhea and fecal incontinence, but the difference with chronic constipation was not statistically significant. The health behaviors subscale was also negatively correlated with chronic diarrhea, chronic constipation, and fecal incontinence, but health factors were negatively related to chronic diarrhea and fecal incontinence and positively related to chronic constipation. The RCS was consistent with the trend of the logistic regression findings. Sensitivity analyses reconfirmed these outcomes.

**Conclusion:**

LE8 is highly associated with chronic diarrhea and fecal incontinence, not with chronic constipation. Encouraging optimization of CVH levels may be beneficial for bowel disorders, and prevention of bowel disorders may enhance CVH.

## 1 Introduction

Bowel conditions, including diarrhea, constipation, and fecal incontinence, are very common in the population. It has been estimated that the prevalence of chronic diarrhea, constipation, and fecal incontinence is as high as 5, 14, and 7%, respectively, in the general population (1, 2). Chronic diarrhea can negatively impact the mental health of patients, leading to a loss of self-confidence and an increased risk of adverse medical outcomes (3, 4). Similarly, chronic constipation affects the quality of life and adds to the financial burden of individuals (5, 6). Fecal incontinence is usually related to cultural stigma, which can cause poor self-image, social isolation, and inconvenience in life, resulting in considerable suffering for patients (7, 8). In spite of this, bowel symptoms are easily overlooked in routine clinical practice, contributing to inadequate or ineffective treatment. The development of diarrhea, constipation, and fecal incontinence is influenced by multiple factors, such as gender, age, physical condition, lifestyle, and diet (9, 10). Therefore, prevention and intervention for intestinal symptoms may be promising in daily life.

The American Heart Association (AHA) introduced Life’s Simple 7 (LS7) to measure cardiovascular health (CVH) in 2010, which includes a well-balanced diet, non-smoking, a healthy body mass index (BMI), a reasonable level of physical activity, blood pressure, fasting blood glucose, and total cholesterol, to better promote health for the population (11). Recently, to better assess CVH, the AHA launched Life’s Essential 8 (LE8), which included sleep quality metrics and upgraded the scoring algorithm compared to the original LS7 (12). The LE8 scoring system captures differences in individuals more accurately and emphasizes the important role of social factors in CVH (13).

The connection between cardiovascular health and gastrointestinal diseases is a complex and increasingly studied field. In recent years, a growing body of research has shown a close interrelationship between cardiovascular health and gastrointestinal diseases, which may be mediated through various mechanisms, particularly inflammatory and metabolic pathways. Cardiovascular diseases (CVDs) and gastrointestinal diseases are both associated with systemic inflammation. It is well known that CVDs are linked to increased levels of inflammatory markers such as C-reactive protein (CRP) (14, 15). Similarly, inflammatory bowel disease (IBD), as a common intestinal disease, is also associated with increased CRP and other inflammatory mediators (16, 17). In addition, dysbiosis is related to an increased risk of CVD and gastrointestinal diseases. Dysbiosis can lead to impaired gut barrier function, thereby increasing the production and systemic inflammation of inflammatory mediators. Short-chain fatty acids (SCFAs), such as butyrate, propionate, and acetate, produced by the fermentation of dietary fiber by gut microbiota, are crucial for maintaining both gut and cardiovascular health. SCFAs have anti-inflammatory effects and can improve gut barrier function, reducing the production and absorption of endotoxins, which lowers the risk of cardiovascular diseases (18). CVDs are linked to abnormalities in lipid metabolism, and the gut plays a key role in lipid metabolism since most cholesterol excretion occurs through the intestines. Gastrointestinal diseases may affect the absorption and excretion of lipids, thereby impacting cardiovascular health.

Currently, no research is available on CVH and gut conditions. Therefore, this study aimed to estimate the relationship between LE8 and diarrhea, constipation, and fecal incontinence, using data from the National Health and Nutrition Examination Survey (NHANES).

## 2 Materials and methods

### 2.1 Study population

This cross-sectional study included nationally representative subjects with consecutive NHANES from 2005 to 2010. These cycles were selected because participants took the bowel health questionnaires during these waves. The NHANES was approved by the National Center for Health Statistics Ethics Review Board, and all participants submitted written informed consent. Of the 31,034 participants in the NHANES from 2005 to 2010, individuals were excluded if they (1) were younger than 20 years of age (*n* = 13,902); (2) had any missing LE8 data (*n* = 5,273); (3) had incomplete bowel health questionnaire data (*n* = 657); and (4) had missing data on covariates, including education, marriage, CRP, CVD, and cancer (*n* = 94). Ultimately, a total of 11,108 subjects participated in the study (Supplementary Figure S1).

### 2.2 LE8 measurement

The LE8 score consists of four health behaviors including diet, physical activity, nicotine exposure, and sleep duration, and four health factors including body mass index/BMI, non-high-density lipoprotein (HDL) cholesterol, blood glucose, and blood pressure (12). Dietary indicators were assessed from 24-h dietary recalls of the subjects using the 2015 Healthy Eating Index (HEI) (19). Physical activity, nicotine exposure, sleep, and history of diabetes and medication used to calculate blood pressure and glucose scores were obtained through a self-report questionnaire. Height, weight, and blood pressure were measured by physical examination. Non-HDL cholesterol, plasma glucose, and hemoglobin A1c were collected from subjects’ blood samples. The specific algorithms for calculating LE8 scores for the metrics in the NHANES data have been reported earlier, where the 8 CVH metrics were scored on a scale ranging from 0 to 100, and the total LE8 score was calculated as an unweighted average of the 8 metrics (Supplementary Table S1) (12, 13). LE8 scores for low, medium, and high CVH participants were 0–49, 50–79, and 80–100, respectively (12). Meanwhile, the same cutoff values were used to divide the health behavior and health factor scores to further explore the relationship between the LE8 subscale and bowel symptoms.

### 2.3 Bowel condition assessment

The Bowel Health Questionnaire was used to identify chronic diarrhea, constipation, and fecal incontinence. Stool consistency was evaluated using the Bristol Stool Form Scale (BSFS) cards (color picture cards with pictures and written descriptors of the seven stool types) and the following written questions: “Please look at this card and tell me the number that corresponds with your usual or most common stool type.” The stool frequency assessment question was “How many bowel movements do you usually have per week?”. Fecal incontinence was measured by questions on the Fecal Incontinence Severity Index, inquiring about the frequency of accidental leakage of gas, mucus, liquid, and solid feces in the last month (20). Diarrhea is defined by having the usual or most common type of stool identified as BSFS type 6 (fluffy pieces with ragged edges, a mushy stool) or BSFS type 7 (watery, no solid pieces) or > 21 bowel movements per week (21). Constipation is considered to have the usual or most common type of stool defined as BSFS type 1 (separate hard lumps such as nuts) or BSFS type 2 (such as sausage, but with lumps) or < 3 bowel movements per week (22). Fecal incontinence is determined as any involuntary loss of mucus, liquid, or solid stool, excluding gas, within the last 30 days (7).

### 2.4 Covariate assessment

Covariates included sociodemographic factors, lifestyle habits, comorbidities, and CRP. Sociodemographics included age, sex (female and male), ethnicity (Mexican American, Non-Hispanic Black, Non-Hispanic White, other Hispanic, and other ethnic groups), education (<high school, high school, and college), marital status (divorced/separated/widowed, married/living with a partner, and never married), and poverty-to-income ratio (PIR) (<1.3, 1.3–3.5, > 3.5, no record). Lifestyle habits refer to drinking status (never, former, and now). Comorbidities included hypertension, CVD, diabetes mellitus (DM), and cancer, where hypertension and DM were confirmed through index measurements, medication use, and self-reporting, and CVD and cancer were ascertained through self-reporting.

### 2.5 Statistical analysis

The appropriate weights were chosen for sample analyses, considering the complexity of the NHANES sampling design. Descriptive statistics were analyzed for baseline characteristics based on different low/moderate/high CVH groups. Continuous variables were displayed as weighted means (standard error) using ANOVA to compare differences between groups; categorical variables were expressed as sample sizes (weighted percentages) using the Rao–Scott *χ*^2^ test to establish differences between groups.

Weighted logistic regression was used to analyze the relationships between LE8 scores and different degrees of CVH and chronic diarrhea, constipation, and fecal incontinence. The crude model did not adjust for any potential confounders. Model 1 adjusted for age, sex, ethnicity, education, marital status, PIR, and alcohol consumption. Model 2 was further adjusted for hypertension, CVD, DM, cancer, and CRP. In addition, the relationships among health behavior, health factor scores, and each of the LE8 scores with bowel conditions were investigated using weighted logistic regression analyses. Stratified analyses by sex, age, marital status, ethnicity, education, PIR, alcohol consumption, hypertension, DM, CVD, and cancer were conducted to examine the relation between LE8 and bowel conditions in different subpopulations. Restricted cubic spline curves (RCS) were used to further validate the connections between LE8, subscale scores, and bowel conditions. Multiplicative interaction tests examined the interaction between the LE8 score and each stratification factor. In sensitivity analyses, we re-analyzed the relationship between LE8/CVH and bowel conditions after excluding all subjects with comorbidities and redefining chronic diarrhea and constipation using stool type rather than stool frequency to ensure the robustness of the findings.

Statistical analyses for this study were undertaken with R version 4.2.2 (R Foundation for Statistical Computing, Vienna, Austria)^1^, and statistical significance was determined using a two-sided *p*-value <0.05.

## 3 Results

### 3.1 Baseline characteristics of participants classified according to the CVH

Table 1 describes the baseline characteristics of the participants. A total of 11,108 U.S. non-institutionalized residents were enrolled in the study, 51.39% of whom were women, with a mean age of 47.39 ± 0.37 years and low, moderate, and high CVHs of 1,452 (10.60%), 7,725 (68.71%), and 1,931 (20.70%), respectively. The prevalence of chronic diarrhea, chronic constipation, and fecal incontinence in the study population was 7.54, 9.27, and 8.42%, respectively. In terms of demographic sociology, participants in the higher CVH group were more likely to be younger, female, Hispanic, more educated, never married, wealthier, and reported lower alcohol consumption. For comorbidities, high CVH participants were less likely to have hypertension, CVD, diabetes, and cancer than those in the low CVH group. Moreover, the higher the CVH level, the lower the CRP. Regarding bowel conditions, people with better CVH had a lower prevalence of chronic diarrhea, chronic constipation, and fecal incontinence.

**Table 1:** Baseline characteristics of the study population.

Variable	Total	Low	Moderate	High	*p* value
No. of participants	11,108	1,452 (10.60)	7,725 (68.71)	1931 (20.70)	
Age, y, mean (SE)	47.39 (0.37)	53.47 (0.53)	48.08 (0.35)	42.00 (0.59)	<0.0001
Age, n (%)					<0.0001
20–39	3,531 (34.86)	207 (5.36)	2,345 (65.39)	979 (29.25)	
40–59	3,768 (40.73)	563 (12.03)	2,641 (69.83)	564 (18.13)	
≥60	3,809 (24.41)	682 (15.68)	2,739 (71.56)	388 (12.76)	
Sex, n (%)					<0.0001
Female	5,596 (51.39)	752 (11.06)	3,690 (64.76)	1,154 (24.18)	
Male	5,512 (48.61)	700 (10.11)	4,035 (72.88)	777 (17.01)	
Race, n (%)					<0.0001
Mexican American	1936 (7.54)	197 (8.32)	1,408 (71.91)	331 (19.76)	
Non-Hispanic Black	2040 (9.75)	397 (17.45)	1,425 (70.66)	218 (11.90)	
Non-Hispanic White	5,804 (73.60)	705 (10.10)	4,002 (68.42)	1,097 (21.49)	
Other Hispanic	928 (4.20)	116 (9.61)	627 (68.21)	185 (22.18)	
Other Race	400 (4.92)	37 (8.82)	263 (64.71)	100 (26.47)	
Education level, n (%)					<0.0001
<High School	2,907 (16.73)	569 (18.22)	2034 (70.39)	304 (11.39)	
High School	2,662 (24.16)	398 (14.12)	1976 (74.89)	288 (10.99)	
>High school	5,539 (59.11)	485 (7.00)	3,715 (65.70)	1,339 (27.30)	
Marital status, n (%)					<0.0001
Divorced/Separated/Widowed	2,447 (17.92)	501 (18.64)	1700 (69.13)	246 (12.23)	
Married/Living with a partner	6,929 (66.51)	792 (9.23)	4,915 (70.16)	1,222 (20.60)	
Never married	1732 (15.57)	159 (7.18)	1,110 (61.99)	463 (30.83)	
Poverty-to-income ratio, n (%)					<0.0001
<1.3	2,875 (16.66)	523 (16.84)	1991 (69.39)	361 (13.77)	
1.3–3.5	4,018 (34.44)	548 (12.48)	2,828 (69.62)	642 (17.91)	
>3.5	3,453 (43.60)	282 (6.76)	2,369 (67.60)	802 (25.64)	
No record	762 (5.31)	99 (10.33)	537 (69.73)	126 (19.94)	
Alcohol status, n (%)					<0.0001
Never	1,422 (10.44)	174 (9.90)	945 (64.49)	303 (25.61)	
Former	2,218 (16.67)	469 (19.08)	1,553 (70.41)	196 (10.51)	
Now	7,468 (72.89)	809 (8.76)	5,227 (68.92)	1,432 (22.32)	
Hypertension, n (%)					<0.0001
No	6,492 (63.74)	370 (4.98)	4,440 (66.30)	1,682 (28.72)	
Yes	4,616 (36.26)	1,082 (20.48)	3,285 (72.94)	249 (6.58)	
DM, n (%)					<0.0001
No	8,371 (80.13)	715 (7.22)	5,879 (68.70)	1777 (24.08)	
Boundary	1,037 (8.77)	149 (13.22)	771 (74.17)	117 (12.61)	
Yes	1700 (11.10)	588 (32.90)	1,075 (64.43)	37 (2.67)	
CVD, n (%)					<0.0001
No	9,861 (91.55)	1,091 (9.14)	6,906 (68.84)	1864 (22.02)	
Yes	1,247 (8.45)	361 (26.43)	819 (67.24)	67 (6.33)	
Cancer, n (%)					0.01
No	10,011 (90.59)	1,292 (10.44)	6,937 (68.46)	1782 (21.10)	
Yes	1,097 (9.41)	160 (12.12)	788 (71.11)	149 (16.76)	
CRP, mean (SE)	0.37 (0.01)	0.65 (0.03)	0.38 (0.01)	0.21 (0.01)	<0.0001
Chronic diarrhea, n (%)					<0.0001
No	10,145 (92.46)	1,268 (10.09)	7,041 (68.51)	1836 (21.40)	
Yes	963 (7.54)	184 (16.83)	684 (71.08)	95 (12.09)	
Chronic constipation, n (%)					0.02
No	9,996 (90.73)	1,281 (10.31)	6,979 (69.04)	1736 (20.66)	
Yes	1,112 (9.27)	171 (13.44)	746 (65.48)	195 (21.08)	
Fecal incontinence, n (%)					<0.0001
No	10,075 (91.58)	1,255 (9.97)	6,989 (68.56)	1831 (21.47)	
Yes	1,033 (8.42)	197 (17.43)	736 (70.32)	100 (12.25)	

Data were presented as weighted percentages or means (95% confidence intervals); Low CVH was defined as a LE8 score of 0 to 49, moderate CVH of 50–79, and high CVH of 80–100.

### 3.2 Relationship between LE8/CVH and bowel conditions

Table 2 displays the outcomes of the weighted logistic regression between LE8/CVH and chronic diarrhea, constipation, and fecal incontinence. In the stepwise adjustment model (Model 2), with each 10-point increase in LE8 score, the prevalence of chronic diarrhea and fecal incontinence in the study population decreased by 16 and 15% (both *p* < 0.0001), respectively, and the prevalence of chronic constipation decreased by 2%, but the difference was not statistically significant (*p* = 0.54). The analysis of CVH and bowel conditions revealed that in the low-moderate-high CVH group, there was a statistically significant dose-decreasing trend in the odds of chronic diarrhea and fecal incontinence (both P for trend <0.05), which remained stable across models, and that there was no statistically significant correlation between CVH and chronic constipation. Sensitivity analyses excluding comorbidities also detected the same trend of LE8/CVH with bowel conditions (Supplementary Table S2).

**Table 2:** Weighted logistics regression showing the relationship between LE8/CVH and bowel conditions.

	Crude model		Model 1		Model 2	
	OR (95%CI)	*p* value	OR (95%CI)	*p* value	OR (95%CI)	*p* value
Chronic diarrhea						
LE8	0.79 (0.74, 0.84)	<0.0001	0.83 (0.77, 0.89)	<0.0001	0.84 (0.77, 0.90)	<0.0001
CVH						
Low	ref		ref		ref	
Moderate	0.62 (0.48, 0.81)	<0.001	0.72 (0.54, 0.95)	0.02	0.75 (0.56, 1.02)	0.06
High	0.34 (0.24, 0.48)	<0.0001	0.43 (0.29, 0.64)	<0.001	0.46 (0.31, 0.70)	<0.001
p for trend		<0.0001		<0.0001		<0.001
Chronic constipation						
LE8	0.97 (0.91, 1.03)	0.32	1.00 (0.93, 1.07)	0.98	0.98 (0.91, 1.05)	0.54
CVH						
Low	ref		ref		ref	
Moderate	0.73 (0.57, 0.92)	0.01	0.86 (0.66, 1.10)	0.22	0.80 (0.61, 1.04)	0.09
High	0.78 (0.59, 1.04)	0.09	0.90 (0.64, 1.27)	0.55	0.81 (0.58, 1.13)	0.20
p for trend		0.25		0.70		0.34
Fecal incontinence						
LE8	0.78 (0.73, 0.82)	<0.0001	0.84 (0.79, 0.89)	<0.0001	0.85 (0.80, 0.91)	<0.0001
CVH						
Low	ref		ref		ref	
Moderate	0.59 (0.47, 0.73)	<0.0001	0.74 (0.59, 0.94)	0.01	0.78 (0.61, 1.00)	0.05
High	0.33 (0.24, 0.45)	<0.0001	0.49 (0.35, 0.68)	<0.001	0.53 (0.37, 0.77)	0.002
p for trend		<0.0001		<0.0001		0.001

Model 1: Adjusted for age, sex, race, marital status, education, poverty-income ratio, and alcohol using.

Model 2: Additionally adjusted for hypertension, diabetes, CVD, cancer and CRP.

LE8, Life’s Essential 8; CVH, cardiovascular health.

Low CVH was defined as a LE8 score of 0 to 49, moderate CVH of 50–79, and high CVH of 80–100.

LE8 score were brought into the logistic regression model for each 10-point increase.

### 3.3 Relationship between health behavior/health factor scores and bowel conditions

The relationships between health behavior, health factor scores, and gut symptoms are illustrated in Table 3. Health behavior score was negatively associated with chronic diarrhea, constipation, and fecal incontinence (all *p* < 0.05), and its low-moderate-high groups exhibited a dose-decreasing relationship with bowel symptoms (all p for trend <0.05). However, the health factor score was negatively associated with chronic diarrhea and fecal incontinence and positively associated with chronic constipation (all *p* < 0.05). Compared to the low health factor score group, higher health factor scores were correlated with a lower prevalence of chronic diarrhea and fecal incontinence but with a higher prevalence of chronic constipation. In addition, the study explored the relationship between bowel symptoms and specific LE8 scores (Supplementary Table S3) and found that chronic diarrhea was negatively correlated with HEI, nicotine exposure, and BMI scores; chronic constipation was negatively related to HEI, physical activity, sleep, and BMI scores; and fecal incontinence was negatively associated with HEI, sleep, and blood glucose scores, after adjusted relevant characters.

**Table 3:** Weighted logistic regression showing the relationship between health behaviors score/health factors score and bowel conditions.

	Crude model		Model 1		Crude model		Model 1	
	OR (95%CI)	*p* value	OR (95%CI)	*p* value	OR (95%CI)	*p* value	OR (95%CI)	*p* value
	Health behaviors score				Health factors score			
Chronic diarrhea								
LE8 subscale score	0.90 (0.86, 0.94)	<0.0001	0.92 (0.88, 0.97)	0.002	0.86 (0.83, 0.90)	<0.0001	0.89 (0.84, 0.94)	<0.001
Classification								
Low (0–49)	ref		ref		ref		ref	
Moderate (50–79)	0.82 (0.66, 1.03)	0.09	0.90 (0.71, 1.15)	0.39	0.73 (0.57, 0.93)	0.01	0.83 (0.62, 1.11)	0.2
High (80–100)	0.61 (0.46, 0.80)	<0.001	0.71 (0.52, 0.96)	0.03	0.46 (0.36, 0.59)	<0.0001	0.58 (0.41, 0.80)	0.002
p for trend		<0.001		0.03		<0.0001		<0.001
Chronic constipation								
LE8 subscale score	0.90 (0.86, 0.93)	<0.0001	0.93 (0.89, 0.98)	0.004	1.09 (1.04, 1.15)	<0.001	1.09 (1.02, 1.16)	0.01
Classification								
Low (0–49)	ref		ref		ref		ref	
Moderate (50–79)	0.85 (0.70, 1.04)	0.11	0.98 (0.81, 1.18)	0.82	1.02 (0.82, 1.26)	0.87	1.16 (0.87, 1.53)	0.3
High (80–100)	0.59 (0.46, 0.76)	<0.001	0.73 (0.56, 0.96)	0.02	1.50 (1.17, 1.91)	0.002	1.54 (1.09, 2.17)	0.02
p for trend		<0.0001		0.02		<0.001		0.01
Fecal incontinence								
LE8 subscale score	0.91 (0.87, 0.95)	<0.0001	0.91 (0.87, 0.96)	<0.001	0.83 (0.80, 0.87)	<0.0001	0.93 (0.88, 0.98)	0.01
Classification								
Low (0–49)	ref		ref		ref		ref	
Moderate (50–79)	0.84 (0.70, 1.01)	0.06	0.90 (0.74, 1.09)	0.27	0.70 (0.55, 0.89)	0.004	0.92 (0.70, 1.22)	0.56
High (80–100)	0.56 (0.45, 0.71)	<0.0001	0.58 (0.45, 0.76)	<0.001	0.41 (0.33, 0.51)	<0.0001	0.75 (0.55, 1.01)	0.06
p for trend		<0.0001		<0.001		<0.0001		0.05

Model 1: Adjusted for age, sex, race, marital status, education, poverty-income ratio, alcohol using, hypertension, diabetes, CVD, cancer and CRP.

LE8, Life’s Essential 8.

Health behaviors score and health factors score were brought into the logistic regression model for each 10-point increase.

### 3.4 Stratified analyses of LE8 with bowel conditions

**Figure 1:**
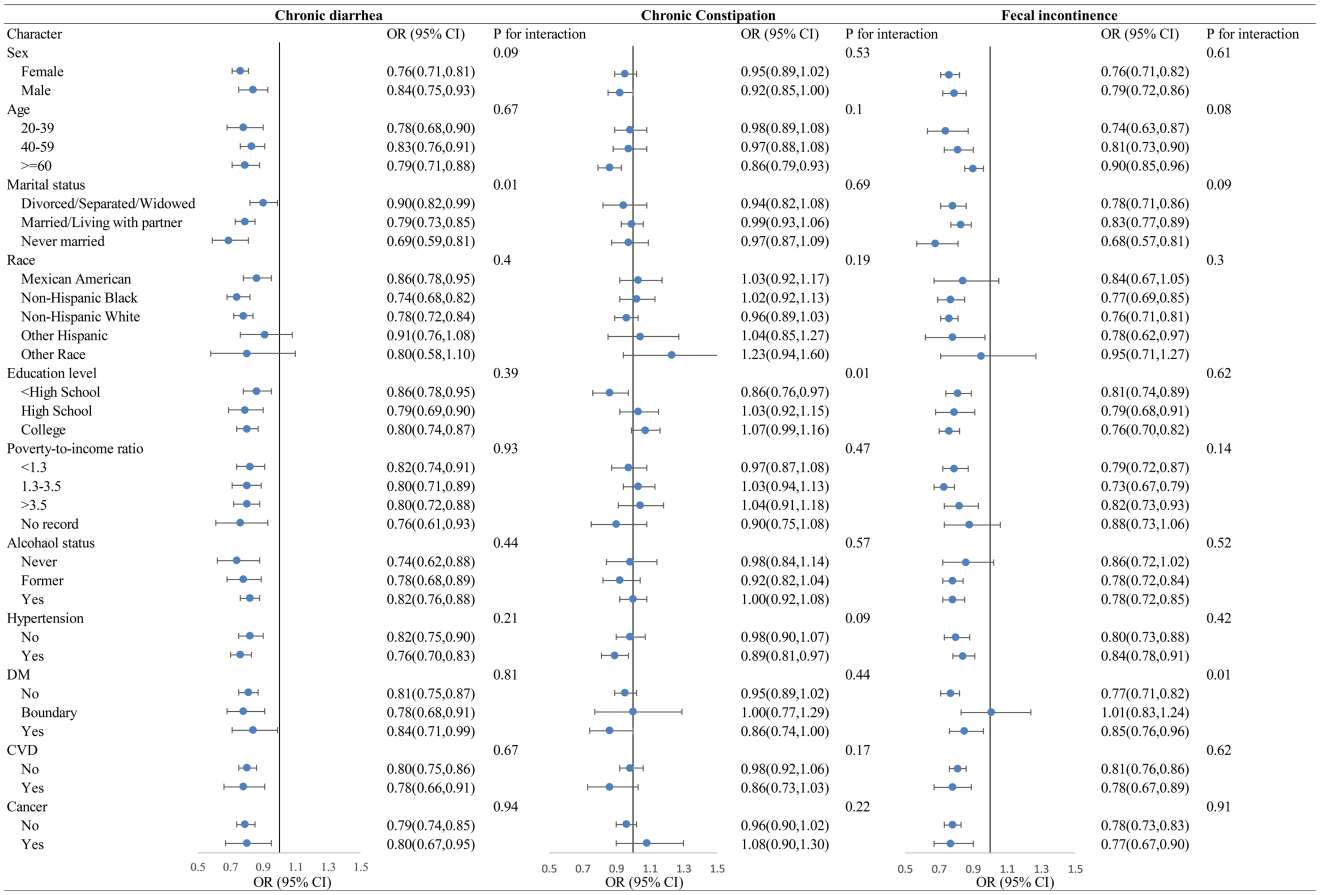
Stratified analysis of bowel conditions and LE8.

As illustrated in Figure 1, stratified analyses suggested that the LE8 was strongly negatively correlated with chronic diarrhea and fecal incontinence, and that the negative correlations remained roughly stable when stratified by sex, age, marital status, ethnicity, education, poverty, drinking status, hypertension, diabetes, CVD, and cancer. The LE8 scores had a statistically significant negative association between chronic constipation only among those ≥60 years of age, <high school education, and hypertension comorbidities.

### 3.5 Restricted cubic spline analysis

**Figure 2:**
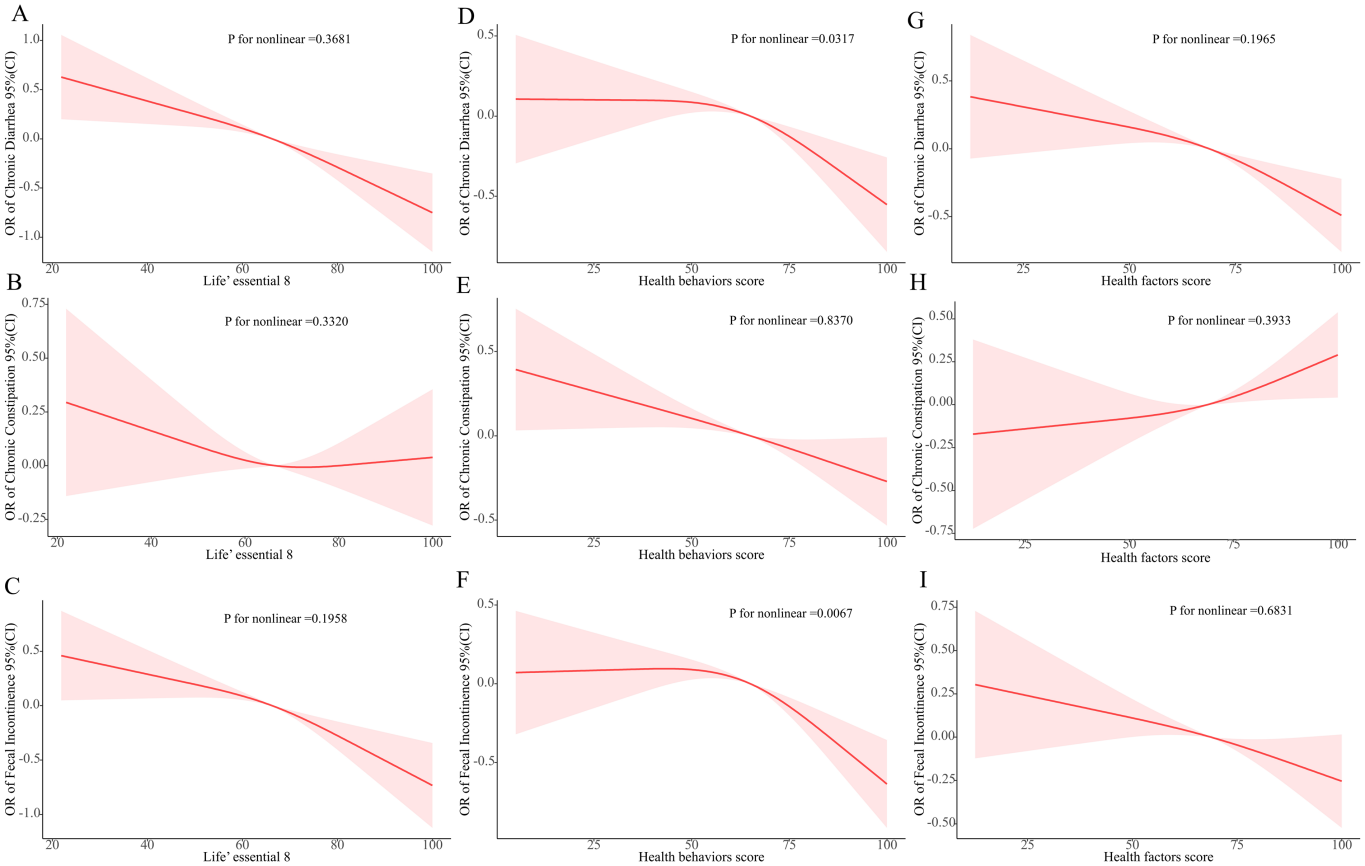
RCS analysis between bowel conditions and LE8.

RCS regression adjusted for all covariates uncovered significant linear negative associations between LE8 and chronic diarrhea, constipation, and fecal incontinence (p for non-linear >0.05, Figures 2A–C). Health behavior scores were non-linearly negatively correlated with chronic diarrhea and fecal incontinence (p for non-linear <0.05, Figures 2D,F) and linearly negatively correlated with chronic constipation (p for non-linear >0.05, Figure 2E). Health factor scores were linearly and negatively associated with chronic diarrhea and fecal incontinence (p for non-linear >0.05, Figures 2G,I) and linearly positively linked to chronic constipation (p for non-linear = 0.3933, Figure 2H). In conclusion, except for chronic constipation, the odds of suffering from chronic diarrhea and fecal incontinence decreased as the LE8, health behavior, and health factors scores increased.

## 4 Discussion

To elucidate the relationship between LE8 scores and bowel symptoms, we performed a cross-sectional analysis of 11,108 participants from the NHANES cohort. The results revealed that higher LE8 scores were associated with a lower prevalence of chronic diarrhea, chronic constipation, and fecal incontinence, but the difference with chronic constipation was not statistically significant. Health behavior and health factor subscales were also negatively correlated with chronic diarrhea and fecal incontinence, and health behaviors were negatively related to chronic constipation; however, health factor scores were positively correlated with chronic constipation. Stratified analyses indicated that the inverse relationship between LE8 scores and chronic diarrhea and fecal incontinence remained stable across stratification factors. Sensitivity analyses showed the relationship between LE8 scores and bowel symptoms, which is consistent with the above results.

To our knowledge, this is the first study to assess the relationship between gut conditions and cardiovascular health. The gut–heart connection can explain the high prevalence of cardiovascular disease in patients with bowel disorders (23). Multiple factors are involved in the gut–heart axis, including increased inflammatory response and oxidative stress (24), altered blood pressure by glucagon-like peptide-1 receptors (25, 26), decreased beneficial metabolites (such as SCFAs), and accumulation of toxic metabolites due to dysbiosis of the intestinal flora (27, 28). Previous studies have indicated that short-chain fatty acids (SCFAs) could serve as a short-chain carbon source for patients with hyperphosphatemia (HF) (28). The antioxidant and mitochondrial stress-protective effects mediated by SCFAs suggest that they can act as nutrients and therapeutic agents for blood vessels (29). Moreover, SCFAs can significantly improve vascular calcification through the reshaping of the gut microbiota (30). Thus, gut health is closely linked to cardiovascular health.

Chronic diarrhea was found to be strongly correlated with LE8 in our study. IBD, irritable bowel syndrome (IBS), functional diarrhea, microscopic colitis, celiac disease, and many other intestinal disorders can lead to chronic diarrhea. Sleutjes et al. found that CVH is mostly suboptimal in patients with IBD, and optimizing CVH may improve the prognosis of patients with IBD (31). A UK BioBank study suggested that patients with celiac disease have fewer traditional cardiovascular risk factors and a higher prevalence of CVH, which may be due to the fact that this study was based on cardiovascular health in LS7 rather than LE8 (32). The HEI-2015 diet score, nicotine exposure score, and body mass index score were inversely associated with chronic diarrhea in the LE8-specific component analysis. Diet and chronic diarrhea have been studied extensively enough. Low FODMAP (fermentable oligo-, di-, monosaccharides, and polyol) diet, gluten-free diet, and vegetable-based modified Mediterranean health diet can alleviate the symptoms of diarrhea, while high-fat and high-protein diets can cause diarrhea (33–37). Smoking is a risk factor for most diseases. Smoking increases the risk of IBD, so anyone with IBD should avoid or quit smoking (38, 39). Smoking expands the incidence of post-infectious IBS (PI-IBS) after acute gastroenteritis, which manifests itself as diarrhea and mixed forms of IBS (40). Smoking is also an independent predictor of postoperative diarrhea after colorectal cancer surgery (41). The relationship between BMI and diarrhea symptoms is controversial. Obese patients with increased small intestinal permeability are more likely to develop IBS-D (34), and obese women have a lower risk of developing microscopic colitis (42), which still requires further study in the future.

Chronic constipation was not statistically associated with LE8, was negatively associated with the health behaviors score, and was positively associated with the health factor score. Lifestyle behaviors were strongly connected to chronic constipation. Low-fiber diets and irregular eating habits are risk factors for constipation, and increased intake of fruits and dietary fiber-rich vegetables may be effective in relieving constipation (43). Physical activity reduces constipation symptoms in older adults by shortening colonic transit time (44). Both too short and too long sleep durations are linked to excessive chronic prevalence (45). An interesting finding was the positive correlation of health factor scores for chronic constipation. We hypothesized that this may be due to the fact that unhealthy factors such as obesity, hyperlipidemia, hyperglycemia, and hypertension may contribute to chronic constipation through the development of the disease itself and the use of therapeutic medications (e.g., antihypertensive drugs) (46).

Fecal incontinence is highly related to LE8. There are fewer studies on fecal incontinence and CVD. Fecal incontinence can be caused by anal sphincter trauma (previous surgery or obstetric injury), intestinal dysfunction (especially diarrhea), rectal urgency, disorders of the pelvic floor anatomy, inflammatory bowel disease, neurological disorders, and chronic diseases (2). Our study discovered that people with healthy behaviors and health factors, especially good diet, sleep, and blood glucose, have a lower chance of developing fecal incontinence, which provides several new entry points into the etiology and prevention of fecal incontinence.

Our findings indicated a robust negative correlation between LE8 and chronic diarrhea and fecal incontinence, which remained consistent across different strata, highlighting a broad impact of cardiovascular health on bowel conditions. The significant correlation with chronic constipation only in specific groups, such as the elderly and those with hypertension, suggested that age and comorbidities might modulate this relationship. The physiological changes associated with aging, including altered gut motility, could partially explain the observed correlations (47). In addition, the use of medications (such as calcium channel blockers) for comorbid conditions such as hypertension can further affect gut function, potentially influencing the risk of chronic constipation (48). The distinct correlation in the elderly also implies that the interplay between cardiovascular health and bowel conditions becomes more nuanced with age. This could be due to age-related shifts in the gut microbiota, which are known to produce metabolites such as trimethylamine N-oxide (TMAO) that are associated with cardiovascular disease risk (49). Variations in lifestyle and dietary habits among the elderly, compared to younger populations, may also play a role in the observed correlations. For instance, habits that promote cardiovascular health, such as moderate alcohol consumption and regular physical activity, could indirectly impact bowel conditions.

Strengths of this study include the use of high-quality NHANES data, a large sample size, adjustment for many important covariates, use of the most recent LE8 to reflect cardiovascular health, and in-depth analysis of the relationship between LE8 and bowel symptoms. Meanwhile, there are some limitations worth addressing. First, the cross-sectional study could not conclude a causal relationship between LE8 and chronic diarrhea, constipation, and fecal incontinence. Second, due to database limitations, bowel conditions in this study were diagnosed based on symptoms from the BHQ, which was highly subjective and may be subject to recall bias. Third, there are many causes related to bowel disorders, such as pancreatic diseases and infectious bowel diseases, but this information could not be analyzed because it was not included in the NHANES database. Fourth, the NHANES data from 2005 to 2010 are somewhat outdated, making it crucial to obtain more recent data to substantiate our findings. Despite these limitations, this is the first study to assess the associations between LE8 and chronic diarrhea, and we believe that it still provides valuable insights into the relationship between cardiovascular health and bowel diseases.

In conclusion, the study found a negative association between the LE8 score and chronic diarrhea and fecal incontinence, but not with chronic constipation. Health behaviors were inversely related to chronic diarrhea, chronic constipation, and fecal incontinence, while health factors were negatively correlated with chronic diarrhea and fecal incontinence and positively correlated with chronic constipation. The findings highlight the strong connection between cardiovascular health and bowel health and suggest that adherence to LE8 may be an effective way of preventing bowel disease, and that prevention of bowel disease may also have the potential to improve cardiovascular health. Further research is needed to fully understand the causal relationship and exact mechanism between LE8 and intestinal diseases.

## Data Availability

Publicly available datasets were analyzed in this study. This data can be found here: https://www.cdc.gov/nchs/nhanes/index.htm.

## Footnotes

^1^http://www.r-project.org
