# Supplementary material for: Association between ideal cardiovascular health and bowel conditions among US adults
Source: Front Nutr. 2024 Nov 7;11:1473531. doi: 10.3389/fnut.2024.1473531 (PMC11580258; doi:10.3389/fnut.2024.1473531)
Supplement: Supplementary file 1 [file Data_Sheet_1.docx]

Supplementary Material

Association between Ideal Cardiovascular Health and Bowel Conditions among US Adults

Yiwen Wang^1^, Zhigang Wang ^1,^ *

*** Correspondence:** Zhigang Wang; wangzhigang198406@163.com

# Supplementary Figures and Tables

## Supplementary Figures

Participants extracted from NHANES

2005-2010 (n=31034)

Excluded:

Participants with age <20 years (n=13902).

Included participants (n=17132)

Excluded:

Participants with missing data on Life’s Essential 8 (n=5273);

Included participants (n= 11859)

Excluded:

Participants with missing values on bowel health questionnaires

(n = 657).

Included participants (n=11202)

Eligible participants for analysis (n=11108)

Excluded:Participants with missing data on education, marriage, C-reactive protein, cardiovascular disease and cancer (n = 94);

**Figure S1. Flowchart of the sample selection from NHANES 2005-2010**

## Supplementary Tables

**Table S1. Definition and scoring approach for the American Heart Association’s Life’s Essential 8 score.** (1)

| Domain | CVH Metric | Measurement | Quantification and Scoring of CVH Metric |
| --- | --- | --- | --- |
| Health Behaviors | Diet | Healthy Eating Index-2015 diet score percentile | Quantiles of DASH-style diet adherence  **Scoring (Population):**  Points Quantile  100 ≥95^th^ percentile (top/ideal diet)  80 75^th^ – 94^th^ percentile  50 50^th^ – 74^th^ percentile  25 25^th^ – 49^th^ percentile  0 1^st^ – 24^th^ percentile (bottom/least ideal quartile) |
|  | Physical activity | Self-reported minutes of moderate or vigorous physical activity per week | **Metric:** Minutes of moderate (or greater) intensity activity per week  **Scoring:**  Points Minutes  100 ≥150  90 120 – 149  80 90 – 119  60 60 – 89  40 30 – 59  20 1 – 29  0 0 |
|  | Nicotine exposure | Self-reported use of cigarettes or inhaled nicotine- delivery system | **Metric:** Combustible tobacco use and/or inhaled NDS use; or secondhand smoke exposure  **Scoring:**  Points Status  100 Never smoker  75 Former smoker, quit ≥5 yrs  50 Former smoker, quit 1 - <5 yrs  25 Former smoker, quit <1 year, or currently using inhaled NDS  0 Current smoker  Subtract 20 points (unless score is 0) for living with active indoor smoker in home |
|  | Sleep health | Self-reported average hours of sleep per night | **Metric:** Average hours of sleep per night  **Scoring:**  Points Level  100 7 – <9  90 9 – <10  70 6 – <7  40 5 – <6 or ≥10  20 4 – <5  0 <4 |
| Health Factors | Body mass index | Body weight (kg) divided by height squared (m^2^) | **Metric:** Body mass index (kg/m^2^)  **Scoring:** Points Level 100 <25  70 25.0 – 29.9  30 30.0 – 34.9  15 35.0 – 39.9  0 ≥40.0 |
|  | Blood lipids | Plasma total and HDL-cholesterol with calculation of non-HDL-cholesterol | **Metric:** Non-HDL-cholesterol (mg/dL)  **Scoring:**  Points Level  100 <130  60 130 – 159  40 160 – 189  20 190 – 219  0 ≥220  If drug-treated level, subtract 20 points |
|  | Blood glucose | Fasting blood glucose or casual hemoglobin A1c | **Metric:** Fasting blood glucose (mg/dL) or Hemoglobin A1c (%)  **Scoring:**  Points Level  100 No history of diabetes and FBG <100 (or HbA1c < 5.7)  60 No diabetes and FBG 100 – 125 (or HbA1c 5.7-6.4) (Pre-diabetes)  40 Diabetes with HbA1c <7.0  30 Diabetes with HbA1c 7.0 – 7.9  20 Diabetes with HbA1c 8.0 – 8.9  10 Diabetes with Hb A1c 9.0 – 9.9  0 Diabetes with HbA1c ≥10.0 |
|  | Blood pressure | Appropriately measured systolic and diastolic blood pressure | **Metric:** Systolic and diastolic blood pressure (mm Hg)  **Scoring:**  Points Level  100 <120/<80 (Optimal)  75 120-129/<80 (Elevated)  50 130-139 or 80-89 (Stage I HTN)  25 140-159 or 90-99  0 ≥160 or ≥100  Subtract 20 points if treated level |

**Reference**

1. Lloyd-Jones DM, Allen NB, Anderson CAM, Black T, Brewer LC, Foraker RE, Grandner MA, Lavretsky H, Perak AM, Sharma G, et al. Life’s Essential 8: Updating and Enhancing the American Heart Association’s Construct of Cardiovascular Health: A Presidential Advisory From the American Heart Association. *Circulation* (2022) 146:e18–e43. doi: 10.1161/CIR.0000000000001078

**Table S2.Sensitivity analysis weighted logistic regression on LE8/CVH and bowel conditions.**

|  | Crude model | | Model 1 | |
| --- | --- | --- | --- | --- |
|  | OR (95%CI) | P value | OR (95%CI) | P value |
| **Chronic diarrhea** |  |  |  |  |
| LE8 subscale score | 0.83(0.75,0.92) | <0.001 | 0.85(0.76,0.96) | 0.01 |
| Classification | 95%CI | P | 95%CI | P |
| Low (0–49) | ref |  | ref |  |
| Moderate (50–79) | 0.79(0.43,1.45) | 0.44 | 0.87(0.46,1.64) | 0.65 |
| High (80–100) | 0.48(0.26,0.88) | 0.02 | 0.56(0.29,1.07) | 0.08 |
| p for trend |  | <0.001 |  | 0.01 |
|  |  | |  | |
| **Chronic Constipation** |  |  |  |  |
| LE8 subscale score | 1.05(0.97,1.15) | 0.22 | 1.07(0.98,1.17) | 0.13 |
| Classification |  |  |  |  |
| Low (0–49) | ref |  | ref |  |
| Moderate (50–79) | 0.79(0.48,1.29) | 0.34 | 0.84(0.50,1.40) | 0.49 |
| High (80–100) | 0.89(0.55,1.45) | 0.64 | 0.95(0.55,1.63) | 0.84 |
| p for trend |  | 0.64 |  | 0.62 |
|  |  |  |  |  |
| **Fecal incontinence** |  |  |  |  |
| LE8 subscale score | 0.82(0.74,0.91) | <0.001 | 0.84(0.76,0.92) | <0.001 |
| Classification |  |  |  |  |
| Low (0–49) | ref |  | ref |  |
| Moderate (50–79) | 0.93(0.51,1.68) | 0.80 | 1.02(0.54,1.93) | 0.94 |
| High (80–100) | 0.51(0.27,0.96) | 0.04 | 0.58(0.31,1.10) | 0.09 |
| p for trend |  | <0.001 |  | 0.001 |

*Crudel model: unadjusted model;

Model 1: Adjusted for age, sex, race, marital status, education, poverty-income ratio, alcohol using, and CRP.

LE8, life’s essential 8;

LE8, health behaviors score and health factors score were brought into the logistic regression model for each 10-point increase.

**Table S3. Weighted logistic regression showing the relationship between each LE8 score and bowel conditions.**

|  | Crude model | | Model 1 | |
| --- | --- | --- | --- | --- |
|  | OR (95% CI) | p | OR (95% CI) | p |
| **Chronic diarrhea** |  |  |  |  |
| HEI-2015 diet score | 0.964(0.943,0.985) | 0.001 | 0.960(0.937,0.985) | 0.003 |
| Physical activity score | 0.972(0.955,0.989) | 0.002 | 0.993(0.975,1.011) | 0.418 |
| Nicotine exposure score | 0.970(0.951,0.989) | 0.003 | 0.971(0.949,0.994) | 0.015 |
| Sleep health score | 0.959(0.930,0.990) | 0.011 | 0.977(0.946,1.008) | 0.134 |
| Body mass index score | 0.928(0.907,0.949) | <0.0001 | 0.941(0.919,0.964) | <0.0001 |
| Blood lipids score | 0.958(0.933,0.985) | 0.003 | 0.970(0.940,1.001) | 0.057 |
| Blood glucose score | 0.929(0.903,0.956) | <0.0001 | 0.999(0.948,1.053) | 0.976 |
| Blood pressure score | 0.955(0.931,0.980) | <0.001 | 0.985(0.957,1.014) | 0.305 |
|  |  |  |  |  |
| **Chronic Constipation** |  | |  | |
| HEI-2015 diet score | 0.947(0.926,0.969) | <0.0001 | 0.954(0.929,0.980) | 0.002 |
| Physical activity score | 0.961(0.946,0.977) | <0.0001 | 0.980(0.964,0.996) | 0.019 |
| Nicotine exposure score | 0.999(0.975,1.023) | 0.912 | 1.007(0.983,1.032) | 0.536 |
| Sleep health score | 0.935(0.912,0.957) | <0.0001 | 0.954(0.928,0.981) | 0.002 |
| Body mass index score | 1.054(1.025,1.085) | <0.001 | 1.063(1.034,1.092) | <0.001 |
| Blood lipids score | 1.029(1.003,1.055) | 0.029 | 1.002(0.973,1.031) | 0.892 |
| Blood glucose score | 1.001(0.970,1.033) | 0.954 | 0.970(0.921,1.020) | 0.224 |
| Blood pressure score | 1.045(1.020,1.071) | <0.001 | 1.032(0.999,1.065) | 0.054 |
|  |  |  |  |  |
| **Fecal incontinence** |  | |  | |
| HEI-2015 diet score | 0.996(0.971,1.022) | 0.761 | 0.968(0.943,0.994) | 0.02 |
| Physical activity score | 0.957(0.941,0.974) | <0.0001 | 0.986(0.968,1.004) | 0.129 |
| Nicotine exposure score | 0.984(0.963,1.005) | 0.129 | 0.976(0.950,1.002) | 0.069 |
| Sleep health score | 0.945(0.914,0.976) | 0.001 | 0.954(0.921,0.988) | 0.011 |
| Body mass index score | 0.953(0.929,0.977) | <0.001 | 0.973(0.948,1.000) | 0.051 |
| Blood lipids score | 0.946(0.918,0.976) | <0.001 | 0.973(0.940,1.008) | 0.126 |
| Blood glucose score | 0.887(0.864,0.910) | <0.0001 | 0.951(0.909,0.994) | 0.028 |
| Blood pressure score | 0.924(0.900,0.949) | <0.0001 | 1.014(0.977,1.053) | 0.44 |

*Crudel model: unadjusted model;

Model 1: Adjusted for age, sex, race, marital status, education, poverty-income ratio, alcohol using, hypertension, diabetes, CVD, cancer and CRP.

LE8, life’s essential 8; HEI, healthy eating index.

Each LE8 score was brought into the logistic regression model for each 10-point increase.
